# Mechanism of antidiabetic effects of *Plicosepalus Acaciae* flower in streptozotocin-induced type 2 diabetic rats, as complementary and alternative therapy

**DOI:** 10.1186/s12906-020-03087-z

**Published:** 2020-09-23

**Authors:** Mohamed-I Kotb El-Sayed, Shaza Al-Massarani, Ali El Gamal, Amina El-Shaibany, Hassan M Al-Mahbashi

**Affiliations:** 1 Department of Biochemistry and molecular Biology, Faculty of Pharmacy, Helwan University, Ain Helwan, Helwan, P.O. Box 11790, Cairo, Egypt; 2 Department of Biochemistry, Faculty of Pharmacy, Ahram Canadian University, Giza, Egypt; 3 Department of Pharmacognosy, Pharmacy College, King Saud University, Riyadh, Saudi Arabia; 4 Department of Pharmacognosy, Faculty of Pharmacy, Mansoura University, Mansoura, Egypt; 5 Department of Pharmacognosy, Faculty of Pharmacy, Sana’a University, Sana’a, Yemen; 6 Department of Forensic Medicine and Clinical toxicology, Faculty of Medicine, Sana’a University, Sana’a, Yemen

**Keywords:** Diabetes, Antioxidant, Metformin, *Plicosepalus Acaciae*, Quercetin, Streptozotocin

## Abstract

**Background:**

Diabetes and its related complications remain to be a major clinical problem. We aim to investigate the antidiabetic mechanistic actions of *Plicosepalus Acaciae* (PA) flowers in streptozotocin (STZ)-induced diabetic rats.

**Methods:**

After diabetes induction, rats were divided randomly into five groups, including: 1) normal control group, 2) diabetic control group, 3) diabetic group treated with 150 mg/kg of ethanolic extract of PA flowers, 4) diabetic group treated with 300 mg/kg of ethanolic extract of PA flowers, and 5) diabetic group treated with 150 mg/kg of metformin. After 15 days of treatment; fasting blood glucose, glycated hemoglobin (HBA1c%), insulin, C-peptide, superoxide dismutase (SOD), catalase, reduced glutathione (GSH), malondialdehyde (MDA), triglyceride (TGs), total cholesterol (Tc), low density lipoprotein cholesterol (LDL-c), very LDL (VLDL), high DLc (HDL-c), tumor necrosis factor-α (TNF-α), and interleukin-6 (IL-6) levels were assessed. Histopathology of pancreas was also assessed.

**Results:**

The results showed that PA flower ethanolic extract significantly reduced blood glucose, HBA1c%, MDA, TGs, Tc, VLDL, LDL-c, TNF-α, and IL-6 levels in a dose-dependent manner. All these parameters were already increased by diabetic induction in the untreated diabetic group. Treatment of diabetic rats with PA flower increased insulin, HDL-c, GSH, catalase, and SOD levels. Histological examination showed that the PA flower caused reconstruction, repair, and recovery of damaged pancreas when compared with the untreated group.

**Conclusions:**

PA flower has a potential role in the management of diabetes as complementary and alternative therapy, due to its antioxidant, anti-inflammatory, hypolipidemic, hypoglycemic and insulin secretagogue effects.

## Background

*Plicosepalus acaciae* (PA) is a perennial, green, semi-parasitic mistletoe belonging to the family Loranthaceae. It is widely distributed in Saudi Arabia and has been traditionally used in folk medicine to treat various diseases, including diabetes [1–5]. PA flower is used in Yemen Republic as a decoction (preparation made by boiling or simmering finely divided flower of the plant in water, usually for 15–20 min and then straining when cool, best used fresh) or as an infusion (preparation made by pouring boiling water onto finely divided flower of the plant and leaving it for some time to sleep before straining without pressing the residue, best used fresh).

Previous investigations on related species of the genus *Plicosepalus* (previously known as *Loranthus*) revealed their efficacy as antidiabetic drugs [6]. Bamane et al. [7] reported that methanolic extracts of *Plicosepalus acacia* (PA) and *P. curviflorus* whole plants show significant antioxidant activity. The results of Aldawsari et al [8] confirm and justify the traditional use of *P. acaciae* and *P. curviflorus* extracts as antidiabetic agents. Solid lipid nanoparticles formulations with high lipid content of the Mistletoes *Plicosepalus acaciae* and *P. curviflorus* possess better antihyperglycemic and antioxidant activities.

Type 2 diabetes is the most common type of diabetes mellitus [9] and characterized by insulin resistance and relatively reduced insulin secretion. Type 2 diabetes can be induced in rats by combining a high-fat diet (HFD) with a low dose of streptozotocin (STZ) [10]. There is evidence that reactive oxygen species (ROS), generated by glucose, play a critical role in the cytotoxicity of STZ [11]. Szkudei [12] concluded that STZ enters the β cell via a glucose transporter (GLUT2) and causes alkylation of DNA. DNA damage induces activation of poly ADP-ribosylation, a process that is leads to NAD+ and ATP depletion, ATP dephosphorylation, superoxide, hydrogen peroxide, and hydroxyl radical’s generation. As a result of the STZ action, β cells undergo the destruction by necrosis and causes diabetogenicity of STZ. ROS inactivate the signal pathway between insulin receptor and the glucose transporter system, thus acting as a mediator of insulin resistance and β-cell dysfunction.

Thus, antioxidants might be beneficial in the treatment of diabetes [13]. Both synthetic and natural antioxidants have been proposed for the prevention and treatment of diabetes mellitus [14]. Many plant species worldwide are antidiabetic due to their hypolipidemic, antioxidant, and anti-inflammatory activities [1, 15].

Many phenolic compounds were isolated from PA (also named *Loranthus Acacia*) such as catechin, quercetin, rutin, gallic acid, methyl gallate, and loranthin (a new flavanocoumarin), showed high antioxidant activities [16]. The phytochemical analysis of PA led to the isolation and characterization of four compounds namely, quercetin 3-O-b-Dglucopyranoside, quercetin 3-O-b-(6-O-galloyl)-glucopyranoside, (−) catechin, and catechin 7-O-gallate [17].

As there is still no cure for type 2 diabetes, the goal of treatment is prevention of insulin resistance, the regeneration of destructed β-cells, and the improvement in complications such as hyperlipidemia, and peripheral neuritis. The aim and the novelty of the current study is to try to find a mechanism of action PA flower extract as an anti-diabetic after qualitative and quantitative detection of anti-diabetic active constituents in two types of extracts. Then linking the role of these active constituents with the antioxidant, hypolipidemic, anti-inflammatory, and β-cells regenerative results of whole extract on STZ-induced diabetic rats using biochemical and histopathological investigations.

## Methods

The primary experimental assessed outcome is to evaluate the antidiabetic and pancreatic protective activities of PA flower while the secondary assessed outcome is to investigate the antioxidant, anti-inflammatory and hypolipidemic activities of PA flower; for elucidation its mechanism of action.

### Chemical and solvents

Chloroform, diethyl ether, methanol, ethanol (80%), ethyl acetate, ferric sulfate, lead acetate, Dil. HNO_3,_ sodium hydroxide, disodium citrate, glacial acetic acid, formic acid, methyl cellulose, isopropanol, xylene, paraffin wax, Hematoxylin-Eosin, and metformin hydrochloride were purchased from ACS, Merck. 10% formalin, and potassium ferrocyanide were purchased from BDH, Ltd. (England). Sulfuric acid was purchased from Farm Italia Carrloerba (Italy). Standard rutin trihydrate from Fluka, no.78095. Standard quercetin from Sigma no. Q4954. Standard gallic acid from Fluka no.91215. Streptozotocin (Sigma Chemical Company, USA) was used to induce diabetes in rats. All other chemicals are purchased from Merk with 99% purity. All chemicals were of analytical grade.

### Apparatus and instruments

TLC aluminum plates silica gel 60F254 [20 × 20 cm, 0.2 mm thickness], TLC scanner 3 with a UV cabinet & Linomat 5, CAMAG, WINCATS Program. ACCU CHEK Performa glucometer (Accu-chek® Advantage, Roche Diagnostic, Mannheim, Germany), Teflon homogenizer (Cole-Parmer, Vernon Hills, IL, USA), light microscope, Buchi Rotavapour R-200, Switzerland, UV-spectrophotometer (Serial No. 340065, Japan), Cotton, pumps, aluminum foil, Whatmann no.1 & 42 filter paper, spectrophotometer (Shimadzu 1200, Japan), and ELISA reader (Humareader Human Company 2106/1682).

### Collection and storage of PA flowers

The collection of plant specimens for scientific purposes in Yemen Republic is legal. The permission was obtained from the Plant Research Committee of Sana’a University. PA flower samples were collected from Taiz City, Republic of Yemen. The flowers were removed from the whole plant during flowering stage in April–May 2017. The plant samples were identified by Dr. Hassan Ibrahim, Department of Botany, Faculty of Science, Sana’a University, Sana’a, Yemen. Voucher specimens (registration code: S011), dated as 20/05/2017, were preserved and stored in the herbarium of Department of Pharmacognosy, Faculty of Pharmacy, University of Sana’a, Sana’a, Republic of Yemen. PA flower was stored in a clean, dry piece of cloth, away from sunlight and moisture to dry, with daily checking.

### Preparation of PA flower extracts

100 g of dried flowers were extracted by maceration and stirring with 1000 ml extraction solvent (ethanol 80% or ethylacetate) at 40 °C for 48 h. All extracts were evaporated by rotavapor under low pressure, washed several times with methanol until obtaining the smallest volume. Then, the extracts were transmitted to an evaporation plates for more evaporation in a water bath at 40 °C until the full extract dryness. After that, the evaporation plates were transmitted to a desiccator containing silica gel till the stably weight. By the end of the extraction steps, we obtained 2 different extracts related to different solvents.

### Phytochemical screening of PA flower extracts

#### Qualitative chemical detection

Simple phenols were detected by FeCl_3_, lead acetate, and Dil. HNO_3_ tests. Glycosides were detected by Borntrager, Legal and Keller- killiani tests. Flavonoids were detected by FeCl_3_, lead acetate, Shinoda, and sodium hydroxide tests [18–21].

#### Quantitative chemical detection by high performance thin layer chromatography (HPTLC)

Standard solutions of rutin, quercetin and gallic acid (1 mg/ml methanol) were prepared. Solutions of ethanolic (80%) and ethylacetate flower extracts (10 mg/ml methanol) were also prepared. All solutions were filtered through Wathmann No.42. The plates of silica gel were activated at 105 °C for 10 min. By using an automatic TLC applicator Linomat 5, 5 μl of each rutin, quercetin, gallic acid standard solutions and extracts solutions were spotted on TLC aluminum plates with silica gel 60 F254, as 10 mm interval between spots, and 10 mm from the plate bottom. The spot components of standards and extracts were eluted and separated on the plates by several mobile phases. After complete liquid diffusion until 15 cm height, the plates were dried at ambient temperature, and scanned at 254, 280, 366 nm by CAMAG Scanner 3. The RF and area under curve (AUC) for each component were calculated at the wavelength giving the maximal optical absorption. The estimated concentrations of quercetin, rutin and gallic acid analogues in the extracts were calculated by rating the AUC of the extract component to the AUC of the known concentration to standard. The experiment was repeated 5 times [22–25].

### Animal

The animals were obtained from the Animal House at the Faculty of Science, Sana’a University, Sana’a, Yemen. All rats were housed in cages (25 × 30 × 30 cm, five rats per cage) under pathogen-free conditions at 22–24 °C, 40–60% relative humidity, and 12 h light/dark cycle. All rats had ad libitum access to standard rodent chow and filtered water and were acclimatized for 2 weeks prior to the initiation of the experiment. All procedures were approved by the Animal Care Committee of Sana’a University and performed according to the “Principles of Laboratory Animal Care” as well as specific national laws where applicable. All experimental protocols and handling of the animals were following the Guide for the Care and Use of Laboratory Animals [26].

Fifty male Wistar rats (2–3 months-old, 150–200 g) were selected for antidiabetic activity study while 36 healthy, young, nulliparous, non-pregnant, female Wistar rats weighing between 83 and 118 g were selected for acute toxicity and dose fixation study,

### Acute toxicity and dose fixation study of PA flower ethanolic extract

Acute oral toxicity was carried out according to the procedure described by the Organization for Economic Cooperation and Development (OECD) [27]. Since studies of LD_50_ value in the literature show that there is usually little difference in sensitivity between the sexes, but females are generally slightly more sensitive. These animals were provided with the same diet for 3 weeks during the acclimatization period prior to the test.

Acute toxicity testing of PA flower ethanolic (80%) extract was performed after fasting the animals for 15 h prior, during which the animals were only provided with water. Following the fasting period, the animals were weighed to determine the dose for each animal. The toxicity of PA flower ethanolic extract was tested using five doses: 250, 500, 1000, 2000, and 2500 mg/kg BW (six rats for each dose). Six control rats without any treatment were kept under the same conditions. The animals were observed continuously during the first hour, and then every hour for the following time intervals (6, 12, and 24 h), and then every 24 h for 3 weeks, to detect any physical signs of toxicity such as writhing, gasping, salivation, diarrhea, cyanosis, pupil size, any nervous manifestations, or mortality.

### Antidiabetic activity of PA flower ethanolic extract

#### Preparation of treatment suspensions

PA flower ethanolic (80%) extract suspension was prepared at doses of 150 and 300 mg/kg by suspending 150 or 300 mg ethanolic extracts of the flower in 2 ml vehicle (0.5% methyl cellulose, MC; a suspending agent). Metformin hydrochloride was prepared at a dose of 150 mg/kg suspended in 2 ml vehicle (0.5% MC).

#### Experimental induction of type 2 diabetes by a high-fat diet (HFD) and streptozotocin (STZ)

The rats were allocated into two dietary regimens; 10 rats were fed normal pellet diet (NPD; 12% calories as fat), and 40 rats were fed high-fat diet (HFD; 58% calories as fat) [28]. The composition of HFD was as described by Srinivasan et al. [10]. After 2 weeks of dietary manipulation, the 40 rats that had been fed HFD were injected intraperitoneally (i.p.) with 35 mg/kg body weight (BW) STZ [29] dissolved in 0.1 M disodium citrate buffer (pH 4.5). Ten days after STZ administration, blood samples were obtained from the tail tip, and glucose levels were determined by the glucometer method (Accu-chek® Advantage, Roche Diagnostic, Mannheim, Germany). Only rats with fasting blood glucose levels of 250–300 mg/dl were employed in the study [30].

#### Experiment design

The fifty rats were divided into 5 groups. The group 1 is the normal control (non-diabetic and receive a 2 ml/kg 0.5% MC daily). After diabetes induction, the diabetic animals were randomly allocated into four groups (*n* = 10) as follows; group 2: diabetic untreated control (receive a 2 ml/kg 0.5% MC daily); group 3: diabetic rats treated with 150 mg/kg PA flower ethanolic extract; group 4: diabetic rats treated with 300 mg/kg PA flower ethanolic extract; and group 5: diabetic rats treated with 150 mg/kg metformin as a reference. The standard drugs and extracts were suspended in 2 ml vehicle (0.5% MC). All treatment was administered for 15 days using an oral gavage (gastric tube). Age-matched healthy rats were used as normal control in Group 1.

#### Blood and tissue sampling for biochemical analysis

Rat blood glucose levels were measured at the beginning of the study (baseline), day 0 (after diabetes induction, before a course of treatment), day 5, day 10, and day 15 after treatments. The fasting blood samples (8 h) were obtained from the rat tail vein under diethyl ether (1.9%) anesthesia (at 80 μl per liter of volume of a container), and fasting blood glucose level was measured using a digital glucometer (Accu-chek® Advantage, Roche Diagnostic, Mannheim, Germany).

On day 16, after 12 h fasting, at the end of the experiments and before surgical operation of the animals for pancreas isolation, morning blood samples were collected from a rat tail vein under diethyl ether (1.9%) anesthesia (at 80 μl per liter of volume of a container). The blood was separated into three aliquots.

The *first* aliquot was whole blood used for determination of glycosylated hemoglobin HbA1c using a Rat HbA1c assay kit (Catalog# 80300; Crystal Chem. USA).

The *second* aliquot was stored in heparinized tubes and the plasma was used for determinations of triglycerides (TGs), total cholesterol (Tc), and high-density lipoprotein cholesterol (HDL-c) concentrations by using a spectrophotometer (Shimadzu 1200, Japan) and commercial kits (Human, Germany, Randox kits). The data are expressed as mg/dL. Very low-density lipoprotein cholesterol (VLDL-c) and LDL-c were estimated as described by Friedewald et al. [31].

The *third* aliquot was allowed to clot at room temperature and centrifuged at 3000 rpm for 15 min. The sera were stored at − 80 °C until use. The sera were analyzed for C-peptide, insulin, tumor necrosis factor (TNF-α), interleukin (IL)-6, and CRP using a RayBio® Rat C peptide EIA Kit (cat number# EIAR-CPE), RayBio® Rat Insulin ELISA Kit (cat number# ELR-Insulin), RayBio® Rat TNF-α ELISA Kit (cat number# ELR- TNF-α), RayBio® Rat IL-6 ELISA Kit (cat number# ELR-IL-6), and RayBio® CRP ELISA Kit (cat number# ELR-CRP), respectively, according to the manufacturers’ protocols (RayBio® Rat, RayBiotech, Norcross, GA, USA). Serum glutamate oxaloacetate transaminase (SGOT) and serum glutamate pyruvate transaminase (SGPT) were determined spectrophotometrically using commercial kits (Spinreact, S.A./S.A.U. Ctra. Santa Coloma, 7 E-17176 Sant Esteve De Bas (GI) Spain).

The markers of oxidative stress and antioxidants were determined in the pancreatic tissue. At the end of the experimental period, the animals were anesthetized under chloroform anesthesia, a midline incision approximately 4 cm in length was made in the abdomen, and the pancreas was dissected out and placed in a normal saline. Next, 100 mg pancreas were homogenized in phosphate-buffered saline (pH 7.4) using a Teflon homogenizer (Cole-Parmer, Vernon Hills, IL, USA). The homogenate was sonicated and centrifuged at 2000×*g* for 10 min. The supernatant was kept at − 80 °C until the spectrophotometric analysis of the MDA level as described by [32] reduced glutathione (GSH) level as described by [33], and activities of superoxide dismutase (SOD) was determined by measuring the inhibition of auto-oxidation of epinephrine at pH 10.2 at 30 °C as described by [34] with modification form by [35] as well as activity of catalase as described by [36] while hydrogen peroxide (H_2_O_2_) was generated and measured by the method described by [37], and nitric oxide (NO) was determined using NO colorimetric assay kit (Roche-Boehringer Mannheim).

Total protein was determined by a colorimetric method using a commercial kit (Spinreact, S.A./S.A.U. Ctra. Santa Coloma, 7 E-17176 Sant Esteve De Bas, Spain) according to the manufacturer’s protocol. Any measurement of each biochemical marker for each sample was repeated three times.

#### Pancreatic tissue sampling for histopathological examination

At end of the experimental period, the last blood sample was collected. Histopathological study of pancreas was carried out at the Department of Pathology, Faculty of Medicine and Health Sciences, Sana’a University, Sana’a, Yemen. At the end of the experimental period, three rats from each group were surgically operated on under diethyl ether (1.9%) anesthesia (at 80 μl per liter of volume of a container). A midline incision ≈ 4 cm in length was made on the abdomen. The pancreas was dissected out and placed in normal saline. Half of the pancreatic tissue (including the tail part) was fixed in PBS containing 10% formalin. The tissues were washed in running tap water, dehydrated in descending grades of isopropanol, and finally cleared in xylene. The tissues were then embedded in molten paraffin wax, cut into transverse 5 μm sections of the mid organ level, and stained with Hematoxylin-Eosin. Histopathological changes in the pancreatic tissues were observed under light microscope 400X-50X [38]. The severity of pancreatitis was determined by the degree of edema, hemorrhage, atrophy, necrosis, and fatty change.

After experiment, the animals were sacrificed using cervical dislocation diethyl ether (1.9%) anesthesia (at 80 μl per liter of volume of a container).

All experiments were performed blindly and impartially by placing a code for each sample related to the group where the necessary analyzes or investigations were performed and recorded, and then the codes are revealed to identify to which group belongs.

### Statistical analysis

Data are expressed as mean ± SEM. Statistical analysis of fasting blood glucose levels were performed using the Two-Way Analysis of Variance (ANOVA) followed by the Bonferroni multiple comparison tests (vs. either normal control and/or vs. diabetic control groups). Statistical analyses for the rest of parameters were performed using the One-Way ANOVA followed by the Bonferroni multiple comparison tests (vs. either normal control and/or vs. diabetic control groups). All analyses, statistical calculations, and the graph were performed using GraphPad Prism Software version 5.0 (GraphPad Software, San Diego, CA, USA). *p* < 0.05 was considered as statistically significant.

## Results

### Phytochemical screening of PA flower extracts

#### The qualitative chemical detection

It demonstrates the presence of flavonoids, glycosides and simple phenols in both studied extracts. The highest estimated concentrations of flavonoids and glycoside were detected in ethanolic extract. Borntrager test was negative in both flower extracts, meaning that anthraquinone glycosides were absents. In contrast, Legal and Keller-Killiani tests were positives, meaning that non-saturated lactone glycosides were present in both flower extracts. Simple phenols were detected only in ethanolic flower extract as ferric chloride and lead acetate tests were positive. In contrast, ferric chloride, lead acetate and diluted nitric acid tests were negatives with ethylacetate extract, meaning the negativity of simple phenols presence. Therefore, the ethanolic extraction by maceration and stirring was better method than ethyl acetate method for the active substance extraction from PA flowers (Table 1).

**Table 1:** Qualitative chemical detection of flavonoids, glycosides and simple phenols in *Plicosepalus acacia* flower extracts

Extraction method	Simple phenol tests	Glycosides test	Flavonoids test
**Ethanol**	FeCl_3_ (+++) Lead acetate (++) Dil.HNO_3_ (+)	Borntrager (−) Legal (++) Keller-Killiani (+++)	FeCl_3_ (+++) Lead acetate (+++) Shinoda (+++) Sodium hydroxide (+++)
**Ethyl Acetate**	FeCl_3_ (−) Lead acetate (−) Dil.HNO_3_ (−)	Borntrager (−) Legal (+) Keller-Killiani (++)	FeCl_3_ (−) Lead acetate (−) Shinoda (+) Sodium Hydroxide (++)

Sign (+) indicates present and sign (−) indicates absent

These two extracts were selected for this plant, after qualitative phytochemical screening was done for many types of extracts including aqueous, chloroform, n-Hexane, ethanol, and ethylacetate (The results of the rest extracts were uploaded as supplementary file). The ethanolic and ethylacetate extracts were have most of active substances (Phenols, glycosides and flavonoids).

#### Quantitative chemical detection by high performance thin layer chromatography (HPTLC)

Quantitative separation of rutin, quercetin and gallic acid standards: The ethyl acetate- glacial acetic acid- formic acid- distilled water (100:11:11:25) was the best mobile phase used for separation of the three standards (rutin, quercetin, gallic acid), and their analogues in the extracts as the best mobile phase. The maximal optical absorptions occurred at 366 nm for rutin (RF: 0.39; area under curve: 10530.2), at 280 nm for quercetin (RF: 0.79; area under curve: 609.9), and at 254 nm for gallic acid (RF: 0.81; area under curve: 2999.8). All previous values were identified in comparison with methanol control, and all area under curves represent a volume of 5 μl of standard solutions (1 mg/ ml).

The separation of the components from The PA flower extracts in conjunction with the separation of the standard have shown that, ethanolic extract contains rutin, quercetin and gallic acid analogues, while ethylacetate extract contains only rutin and quercetin without gallic acid. The ethanolic extract contains rutin and quercetin at highest concentrations than ethylacetate extract (Table 2).

**Table 2:** HPTLC analysis for quantitative separation of rutin, quercetin and gallic acid in *Plicosepalus acacia* flower extracts

Type of the sample	Concentration of rutin (366 nm)			Concentration of quercetin (280 nm)			Concentration of gallic acid (254 nm)		
	R_**f**_	Area	g%	R_**f**_	Area	g%	R_**f**_	Area	g%
**PA Ethanolic Extract**	0.39	14,600.4	3.147	0.79	1750.6	6.515	0.82	927.5	0.701
**PA Ethyl Acetate Extract**	0.42	4860.6	1.047	0.82	299.1	1.113	–	–	–

### Toxicity study

All doses of PA flower ethanolic extract were safe and non-toxic at doses up to 2.5 g/kg BW, with no sign of toxicity during the entire experimental period and no deaths were reported.

### Effect of PA flower on oxidative stress and levels of antioxidant markers

In untreated diabetic control rats, there was a significant reduction in all components of the cellular antioxidant defense system (SOD, catalase, and GSH). Oral administration of PA flower ethanolic extracts significantly increased SOD, catalase and GSH levels in a dose-dependent manner than that of the Met-150 treated groups, with an insignificant difference with that of the normal non-diabetic control group (Table 3). However, the untreated diabetic rats showed a significant increase in MDA and H_2_O_2_ levels compared to that of the normal control. PA flower ethanolic extracts at 150 and 300 mg/kg significantly decreased MDA and H_2_O_2_ levels compared to that of the untreated diabetic rats, whereas the difference in MDA and H_2_O_2_ levels between the treated diabetic groups and normal control groups was insignificant (Table 3). Untreated diabetic rats showed a significant decrease in NO level compared to that of the normal control, but PA flower ethanolic extract at 300 mg/kg significantly increased NO level, whereas PA flower ethanolic extract at 150 mg/kg exhibited no significant effect on NO level (Table 3). These free radical scavenging activities are possibly due to PA flower contents of quercetin, rutin, and gallic acid, which could restore and regulate the activities of catalase, superoxide dismutase and glutathione peroxidase.

**Table 3:** Effect of PA flower ethanolic extracts on SOD, GSH, MDA, NO, and H_2_O_2_ in pancreatic tissue of diabetic rats

Groups/Treatment/Dose (***n*** = 10)	SOD (Unit/mg protein)	Catalase (IU/mg protein)	GSH (μmole/mg protein)	MDA (μmole/mg protein)	NO (μmole/L)	H_**2**_O_**2**_ (μmole/mg protein)
**Normal Control/1 ml/kg**	109.9 ± 1.41	9 ± 0.36	65.5 ± 1.16	0.48 ± 0.033	1.045 ± 0.02	10.6 ± 0.40
**Diabetic Control/1 ml/kg**	83.3 ± 1.37^∗∗∗^	4 ± 0.36^∗∗∗^	15.2 ± 1.14^∗∗∗^	12.9 ± 0.60^∗∗∗^	0.45 ± 0.02^∗∗∗^	13.6 ± 0.42^∗∗∗^
**Diabetic-PA/150 mg/kg**	93.9 ± 2.09^∗∗∗, ###^	6.57 ± 0.4^∗∗∗, ###^	60.6 ± 1.6^**a,** ###^	1.55 ± 0,090^**a,** ###^	0.31 ± 0.01^∗∗∗, ###^	11.7 ± 0.42^**a,** #^
**Diabetic-PA/300 mg/kg**	119.4 ± 0.92^∗∗∗, ###^	7.65 ± 0.27^**a,** ###^	64.5 ± 2.07^**a,** ###^	0.51 ± 0.027^**a,** ###^	0.94 ± 0.02^∗∗∗, ###^	9 ± 0.36^**a,** ###^
**Diabetic-Met/150 mg/kg**	111.1 ± 1.61^**a,** ###^	5.59 ± 0.26^∗∗∗, #^	63.6 ± 1.31^**a,** ###^	1.58 ± 0.05^**a,** ###^	0.81 ± 0.02^∗∗∗, ###^	9 ± 0.37^**a,** ###^

Values are expressed as mean ± SEM; *n* = 10. **∗∗∗** *p* < 0.001 significant vs. Normal Control. **#** *p* < 0.0**5, ###** *p* < 0.001 significant vs. Diabetic Control, **a**; non-significant vs. Normal Control, *SOD* Superoxide dismutase; *GSH* Reduced glutathione; *MDA* Malonaldehyde; *NO*, Nitrous oxide; *H*_*2*_*O*_*2*_ Hydrogen peroxide; *PA Plicosepalus Acacia*; *Met* Metformin

### Effect PA flower on lipid profile

Diabetic rats treated with the PA flower ethanolic extracts showed a significant decrease in Tc, TGs, LDL-c, and VLDL levels in a dose dependent manner compared to those of the untreated diabetic control group. The levels of LDL-c in the group treated with 300 mg/kg flower ethanolic extract were similar to those of the normal non-diabetic control group and/or metformin treated group, but the hypocholesterolemic effect of PA-300 is stronger than that of Met-150 (Table 4). HDL level in rats treated with PA flower ethanolic extracts significantly increased in a dose-dependent manner compared with that of the untreated diabetic control and/or metformin-treated group (Table 4). This hypolipidemic effects possibly due the antioxidant effects of quercetin.

**Table 4:** Effect of PA flower ethanolic extracts *o*n TGs, Tc, LDL-c, HDL-c, and VLDL in diabetic rats

Groups/Treatment/Dose (***n*** = 10)	TGs (mg/dl)	Tc (mg/dl)	VLDL (mg/dl)	LDL-c (mg/dl)	HDL-c (mg/dl)
**Normal Control/1 ml/kg**	103 ± 1.0	81.10 ± 2.12	34 ± 0.88	17.10 ± 0.56	20 ± 0.63
**Diabetic Control/1 ml/kg**	216.5 ± 1.62^∗∗∗^	129.9 ± 1.9^∗∗∗^	79.4 ± 1.75^∗∗∗^	21 ± 0.85^∗∗^	9 ± 0.36^∗∗∗^
**PA-Diabetic/150 mg/kg**	175 ± 1.86^∗∗∗, ###^	94.5 ± 1.14^∗∗∗, ###^	50 ± 1.14^∗∗∗, ###^	18.10 ± 0.56^**a, b**^	21 ± 0.97^**a,** ###^
**PA-Diabetic/300 mg/kg**	151.8 ± 1.25^∗∗∗, ###^	63.2 ± 1.18^∗∗∗, ###^	40.6 ± 0.92^∗∗, ###^	15.4 ± 0.45^**a,** ###^	30.8 ± 0.71^∗∗∗, ###^
**Met-Diabetic/150 mg/kg**	118.4 ± 1.75^∗∗∗, ###^	59.7 ± 0.95^∗∗∗, ###^	50.6 ± 1.10^∗∗∗, ###^	18.3 ± 0.95^**a, b**^	19.10 ± 0.76 ^**a,** ###^

Values are expressed as mean ± SEM; *n* = 10. **∗∗** *p* < 0.01**, ∗∗∗** *p* < 0.001 significant vs. Normal Control. **###** *p* < 0.001 significant vs. Diabetic Control, **a**; non-significant vs. Normal Control, **b**; non-significant vs. Diabetic Control. *TGs* Triacylglycerol; *Tc* Total cholesterol; *VLDL* Very LDL; *LDL-c* Low density lipoprotein cholesterol; *HDL-c* High density lipoprotein cholesterol *PA Plicosepalus Acacia*; *Met* Metformin

### Effect PA flower on levels of inflammatory markers

In the present study, both SGOT and SGPT enzyme activities were used as indicators of hepatic damage. In comparison with the non-diabetic normal rats, the untreated diabetic rats showed increased activities of serum SGOT and SGPT. PA flower ethanolic extract treatment effectively reduced SGOT and SGPT level (in a dose dependent manner) than those of the untreated diabetic control group (Table 5). CRP, TNF-α, and IL-6 are markers whose blood level increase in response to inflammation. Animals treated with PA flower ethanolic extracts at both doses showed significant decrease in CRP, TNF-α, and IL-6 levels than untreated or Met-150 treated groups (Table 5). The free radical scavenging activities of PA flower could minimize the inflammatory consequences in STZ-induced diabetic rats.

**Table 5:** Effect of PA flower ethanolic extracts *o*n SGOT, SGPT, CRP, TNF-α, and IL-6 in diabetic rats

Groups/Treatment/Dose (***n*** = 10)	SGOT (Unit/L)	SGPT (Unit/L)	CRP (mg/dl)	TNF-α (pg/ml)	IL-6 (pg/ml)
**Normal Control/1 ml/kg**	66.5 ± 2.0	50.1 ± 1.7	31.6 ± 1.3	32.6 + 1.3	15 + 0.81
**Diabetic Control/1 ml/kg**	248.6 ± 4.3***	153.2 ± 2.1***,	94 ± 1.6***,	49.3 + 1.3***,	28.9 + 1.2***,
**Diabetic-PA/150 mg/kg**	153.1 ± 2.5***^, **###**^	72.2 ± 2.5***^, **###**^	43.4 ± 0.92***^, **###**^	37.3 + 0.97*^, **###**^	16.8 + 0.94^**a**, **###**^
**Diabetic-PA/300 mg/kg**	116.6 ± 2.4***^, **###**^	62 ± 1.4**, ^**###**^	39 ± 0.96**^, **###**^	34.7 + 0.93^**a, ###**^	15.7 + 0.86 ^**a**, **###**^
**Diabetic-Met/150 mg/kg**	178.4 ± 2.9***^, **###**^	100.7 ± 2.1***^, **###**^	54.4 ± 1.4***^, **###**^	39.8 + 0.84***^,**###**^	19.9 + 0.82**^,**###**^

Values are expressed as mean ± SEM; *n* = 10. **∗** *p* < 0.05, **∗∗** *p* < 0.01**, ∗∗∗** *p* < 0.001 significant vs. Normal Control, **###** *p* < 0.001 significant vs. Diabetic Control, **a**; non-significant vs. Normal Control; *SGOT* Serum glutamic oxaloacetic transaminase; *SGPT* Serum glutamic pyruvic transaminase, *CRP* C-reactive protein; *TNF-α* Tumor necrosis factor-alpha; *IL-6* Interlukin-6; *PA Plicosepalus Acacia*; *Met* Metformin

### Effect of PA flower on fasting blood glucose, HbA1c %, insulin, and C-peptide levels

Injection of STZ after 2 weeks of HFD induced a significant increase in the blood glucose and glycated hemoglobin levels compared to the normal control group (Tables 6 and 7). The current study showed a significant hypoglycemic effect of ethanolic extract of PA flowers in diabetic rats in a dose-dependent manner like those of the metformin-treated rats. HbA1c% was significantly reduced in the metformin treated group than PA-300 treated group, and in the PA-300 treated group than PA-150 treated group. The significant decrease in serum insulin level in the untreated diabetic rats was successfully reversed upon treatment with metformin than PA-300 and with PA-300 than PA-150. All treatment regimens showed an insignificant increase in serum C peptide level compared to that of the untreated diabetic rats (Tables 6 and 7). These results indicate a hypoglycemic activity and regenerative effect of PA flower on pancreatic islets, that possibly due to a higher content of quercetin and rutin in this flower. The antioxidant activities and hypolipidemic effects of these constituents corrects the insulin resistance.

**Table 6:** Effect of PA flower ethanolic extracts on an FBG level in diabetic rats

Groups/Treatment/Dose (n = 10)	Effect on FBG (mg/dl) during treatment period (in days)				
	Baseline	0	5	10	15
**Normal Control/1 ml/kg**	90.10 ± 0.56	89.10 ± 0.64	87.50 ± 0.63	88.6 ± 0.56	86.50 ± 0.92
**Diabetic Control/1 ml/kg**	89.10 ± 0.64^a^	307.3 ± 2.56^***^	303.9 ± 2.45^***^	304.3 ± 2.20^***^	302 ± 1.88^***^
**Diabetic-PA/150 mg/kg**	87.5 ± 0.5^***, **b**^	306.1 ± 1.28^***, **b**^	281.3 ± 1.17^***, ###^	251.4 ± 1.22^***,###^	214.7 ± 3.01^***,###^
**Diabetic-PA/300 mg/kg**	89.10 ± 0.45^a,**b**^	289.9 ± 1.02^***,###^	264.4 ± 1.14^***,###^	229.5 ± 1.08^***,###^	160 ± 1.61^***,###^
**Diabetic-Met/150 mg/kg**	91.3 ± 0.65^a, ##^	279.6 ± 0.96^***,###^	239.9 ± 0.97^***,###^	189.7 ± 0,93^***,###^	148.6 ± 1.05^***,###^

Values are expressed as mean ± SEM; *n* = 10. **∗∗∗** *p* < 0.001 significant vs. Normal Control. **##** *p* < 0.01, **###** *p* < 0.001 significant vs. Diabetic Control, **a**; non-significant vs. Normal Control, **b**; non-significant vs. Diabetic Control. *FBG* Fasting blood glucose; *PA Plicosepalus Acacia*; *Met* Metformin

**Table 7:** Effect of PA flower ethanolic extracts *o*n HBA1c %, Insulin, and C-peptide in diabetic rats

**Groups/Treatment/Dose** **(*n*** **= 10)**	**HBA1c (%)**	**Insulin (mlU/L)**	**C-Peptide (ng/ml)**
**Normal Control/1 ml/kg**	4.4 ± 0.02	35.2 ± 0.46	0.98 ± 0.016
**Diabetic Control/1 ml/kg**	10.8 ± 0.12^***^	18.30 ± 0.36^***^	0.45 ± 0.012^**a**^
**Diabetic-PA/150 mg/kg**	7.5 ± 0.19^***, ###^	25.30 ± 0.47^***, ###^	0.66 ± 0.011^**a, b**^
**Diabetic-PA/300 mg/kg**	6.12 ± 0.11^***, ###^	30.8 ± 0.48^***, ###^	0.86 ± 0.011^**a, b**^
**Diabetic-Met/150 mg/kg**	4.8 ± 0.10^**a,** ###^	31.9 ± 0.43^***, ###^	0.90 ± 0.009^**a, b**^

Values are expressed as mean ± SEM; *n* = 10. **∗∗∗** *p* < 0.001 significant vs. Normal Control. **###***p* < 0.001 significant vs. Diabetic Control, **a**; non-significant vs. Normal Control, **b**; non-significant vs. Diabetic Control. *HBA1c* Glycosylated hemoglobin; *PA Plicosepalus acacia*; *Met* Metformin

### Histological effects PA flower on pancreas

**Fig. 1:**
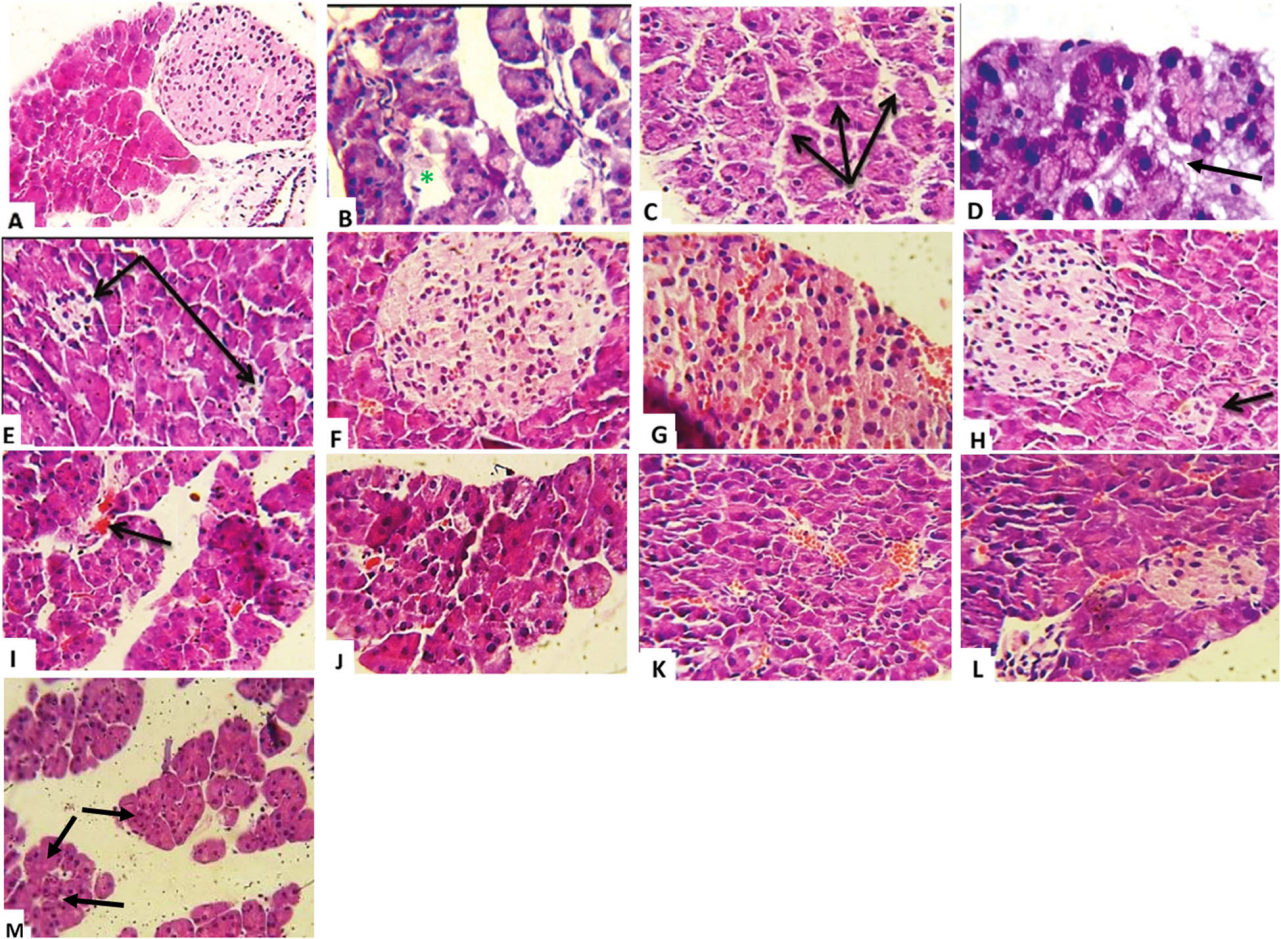
Histological effects of normal saline [**b**, **c**, & **d**], PA flower ethanolic extract (150 mg/kg) [**e**, **f**, & **g**], PA flower ethanolic extract (300 mg/kg) [**h**, **i**, & **j**] and Metformin (150 mg/kg) [**k**, **l**, & **m**] treatments on pancreatic sections in streptozotocin induced diabetic rats in comparison with non-diabetic healthy pancreatic section [**a**]. These figures were showed a normal architecture of pancreatic acini & normal islet of Langerhans in non-diabetic control [**a**], while a reduction in β-cell number i.e., destruction of the islet of Langerhans (*) [**b**] with a necrosis in a pancreatic acini (arrows) [**c**], fatty changes in pancreatic acini (interstitial vaculation ➔ arrow) **[d**] in pancreas of normal saline treated diabetic rats. Moreover, there was a reduction in the size of pancreatic islets; atrophied islets of Langerhans (arrows) [**e**], with a slight hemorrhage in the islets of beta cells (red color) [**f** & **g**] in pancreas of PA (150 mg/kg) treated diabetic rats. A reduction in the size of islets; atrophied islet of Langerhans (arrow) [**h**], with a slight hemorrhage in the pancreatic acini (arrow) [**i**], and a recovering from necrosis in the pancreatic acini [**j**] of PA (300 mg/kg) treated diabetic rats, while a hemorrhage in a pancreatic acini congestion (red color) [**k**], with a reduction in number of beta cells, hemorrhage [**l**], and slight minimal necrotic cells (arrows) [**m**] in the pancreatic acini of Metformin (150 mg/kg) treated diabetic rats (H & E 400X; Scale bar = 50 μm)

Figure 1 illustrates the representative photographs of thin sections of pancreas stained with Hematoxylin-Eosin (H&E, 400×). Histology of the pancreas of normal non-diabetic rats showed no pathological changes, with the normal architecture of pancreatic acini & islet of Langerhans (Fig. 1a).

The pancreas of untreated diabetic rats showed pathological changes such as a reduction in β-cell number; i.e., destruction or depletion of the islet of Langerhans (*) (Fig. 1b), focal area degeneration, congestion, and necrosis in the pancreatic acini (arrows) (Fig. 1c), as well as fat infiltration in the pancreatic acini (interstitial vacuolation; arrow) (Fig. 1d), hemorrhage, arteriosclerosis, and inflammations in both islet and pancreatic acini, as well as cellular infiltration with lymphocytes and mononuclear cells, along with the loss of normal architecture of pancreatic β-cells without any indication of recovery compared to those of the normal tissue (Fig. 1a).

The rats treated with PA flower ethanolic extracts showed significant improvement in cellular architecture as observed by the restoration of normal cellular population size in the islets, along with hyperplasia. Diabetic rats treated with the PA flower ethanolic extract (150 mg/kg) showed preserved islet cells, with minimal reduction in the size of pancreatic islets and atrophied islets of Langerhans (arrows) (Fig. 1e), as well as a slight hemorrhage in the β-cells (showed by red color) (Fig. 1f & g), which generally showed an improvement than that in the untreated diabetic rats. A further dose-dependent improvement was observed in diabetic rats treated with PA flower ethanolic extract (300 mg/kg), including more prominent islet cells, which indicated an improvement in the architecture of the pancreas. This panel showed a minimal reduction in the size of islets and atrophied islet of Langerhans (arrow) (Fig. 1h), with a slight hemorrhage in the pancreatic acini (arrow) (Fig. 1i), and a recovery from necrosis in the pancreatic acini (Fig. 1j). Treatment with PA flower ethanolic extract for 15 days after diabetes-induction resulted in pancreatic morphologies resembling those of normal rats. The amelioration of β-cells, increased vasculature, hypertrophy and hyper-cellularity, along with increased blood vessel in pancreatic islets in these rats reached near normal morphology of pancreatic islets in a dose dependent manner. Meanwhile, hemorrhage and congestion in the pancreatic acini (red color) (Fig. 1k), reduction in β-cells number, hemorrhage (Fig. 1l), and low count of necrotic cells (arrows) (Fig. 1m) without any recovery in the pancreatic acini were observed in metformin (150 mg/kg)-treated diabetic rats.

## Discussion

In the current study, the aim of phytochemical screening was to find the better extraction method of active components from PA flowers, especially those which are known to reduce blood sugar. These active components include flavonoids like quercetin, flavonoid glycosides like rutin, and simple phenols represented by gallic acid. Qualitative detection in the current study, demonstrates the presence of highest concentration of flavonoids, glycosides and simple phenols only in ethanolic flower extract. This may be due to the ability of the ethanol 80% solvent to extract lipophilic and hydrophilic ingredients. Also, we adopt the qualitative detection tests and the HPTLC method to perform the comparison between two different kinds of extracts, and then to identity the best one. The separation of the components from The PA flower extracts in conjunction with the separation of the standard have shown that, ethanolic extract contains rutin, quercetin and gallic acid analogues in highest amounts. These results were the rational reasons for the choosing ethanolic (80%) extract over ethyl acetate extract for assessment of antidiabetic activity of PA flowers. The present study was limited to studying the effect of the whole PA flower extract, and not on its individual active constituents.

These results agree with previous phytochemical studies that indicate that flavonoids, such as catechin, quercetin, rutin, gallic acid, methyl gallate, and loranthin, a new flavanocoumarin, are the major active constituents of PA that contribute to its high radical scavenging activity [16, 17, 39].

Diabetes mellitus is the most common chronic disease that affects the endocrine system. It is a group of metabolic disorders involving carbohydrate, fat, and protein metabolism and characterized by chronic hyperglycemia. It causes various complications, such as metabolic, microvascular, macrovascular, and inflammatory complications due to the adverse effect of increased oxidative stress [40].

Although PA and their isolated constituents have long been used in folk medicine as a remedy for diabetes mellitus [2], their mechanism of action is yet to be proven by any valid research. Therefore, it is important to identify the antioxidant, hypolipidemic, and anti-inflammatory potential of PA flower ethanolic extracts for the treatment of diabetes and its associated complications.

### Antioxidant effects of PA

Oxidative stress caused by increase in intracellular ROS and/or a decrease in antioxidant levels are major factors in the onset and development of diabetes. Furthermore, ROS inactivate the signaling pathway between the insulin receptor and the glucose transporter system, thus acting as a mediator of insulin resistance and β-cell death during the progressive deterioration of glucose tolerance and development of type 2 diabetes. Therefore, antioxidants might be beneficial in the treatment of diabetes and ameliorating its complications [26, 41, 42]. The synthetic antioxidant compounds have shown toxic and/or mutagenic effects, whereas natural antioxidants from natural sources have beneficial effects in preventing chronic diseases, such as diabetes, with fewer side effects [43].

The current results in the same line with many studies that established the antioxidant efficacy of the PA extract or its pure isolates by its marked effect on antioxidant enzymes [7], which correlated to several activities including hepato-protective [44], neuroprotective [45], and anti-diabetic [14]. The antioxidant effect of PA can improve the β-cell repair and reduce insulin resistance [46]. The current results were supported by many studies which stated that flavonoids as major active constituents of PA, such as, quercetin, rutin, and gallic acid were present and showed significant free radical scavenging activity and can restore and regulate the activities of catalase, superoxide dismutase and glutathione peroxidase [16, 47].

### Hypolipidemic effects of PA

A previous study showed that insulin activates lipoprotein lipase resulting in normal triacylglycerol and increased HDL levels [48]. In STZ-induced diabetes, insulin drop caused the failure of lipoprotein lipase to perform normal physiological functions, which leads to hyperlipidemia and hypercholesterolemia.

The current study showed that PA flower ethanolic extracts acted as an antioxidant and insulin secretagogue from the remnant beta cells, so inducing the response of plasma lipoprotein lipase to insulin action, therefore lowering the TGs and Tc levels. Another illustration for hypolipidemic and hypocholesterolemic effects of PA flower ethanolic extracts, is possibly due to its ability to decrease the 3-hydroxy-3-methyl-glutaryl coenzyme A reductase activity by reducing the NADPH required for fatty acid and cholesterol synthesis or by its ability to inhibits lipoxygenases due to its contents of quercetin and rutin so can decrease the level of cholesterol and LDL in diabetic rats [49, 50].

### Anti-inflammatory effects of PA

Insulin deficiency in diabetic patients leads to increased levels of amino acids in the blood, and it will lead to an increase in transaminase activity [51]. So, the increased level of liver function enzymes; SGPT and SGOT in serum is not only used for the identification of liver damage but also for the metabolic syndrome diabetes mellitus; the liver is one of the organs that is affected by diabetes [52]. Many studies confirmed that diabetes induction in animals caused tissue lipid peroxidation and hyperlipidemia in many tissues, also caused secretion and release of liver enzymes to circulation [51]. The current results showed that oral administration of PA flower ethanolic extracts for 15 days induced a significant decrease in the activity of these enzymes in diabetic rats, suggesting that PA has a hepato-protective effect and ameliorates alteration in lipid metabolism. This in line with the results of [53], which showed that quercetin as a constituent of PA could alleviate diabetic symptoms and reduce the disturbance of hepatic gene expression in streptozotocin-induced diabetic mice.

CRP, TNF-α, and IL-6 are markers that increased in the blood in response to inflammation. In the present study, significant changes were observed in the three parameters. When diabetic rats received PA flower ethanolic extracts, there was a significant decrease in their levels. PA flower ethanolic extracts reduce the level of CRP production probably by reducing the level of plasma concentration of interleukin-6 and free radical scavenging activities, minimizing the inflammatory consequences in STZ-induced diabetic rats. It should be noted that antioxidants are also able to ameliorate liver damage [54].

In the current study and in regarding the inhibitory effect of metformin on inflammatory cytokines and its amelioration of liver function, Al-Hashem and his colleagues [55], concluded that metformin protects against thioacetamide-induced hepatic injuries in rats, which is associated with the inhibition of mammalian target of rapamycin (mTOR)–hypoxia inducible factor (HIF)-1α axis and profibrogenic and inflammatory biomarkers; thus, may offer therapeutic potential in humans.

### Hypoglycemic and insulin secretagogue effects of PA

PA is widely distributed in Saudi Arabia and the Republic of Yemen, and commonly used as a traditional medicine for the treatment of diabetes [1]. In the current study the ethanolic extracts of PA flower showed a hypoglycemic effect on diabetic rats in a dose-dependent manner and decreased their blood glucose level from high to normal, like the effect of metformin. The significant decrease of insulin was successfully reversed in the diabetic rats upon treatment with metformin, PA-300, and PA-150. These results agreed with many studies that confirm either the hypoglycemic effect or support the use of PA in the folk medicine as an antidiabetic herb [1, 2, 4, 5, 56].

### Pancreatic protective and repairing effects of PA

Szkudei [12] concluded that STZ enters the β cell via a glucose transporter (GLUT2) and generate many ROS, that causes DNA damage. The generated ROS inactivate the signal pathway between insulin receptor and the glucose transporter system, thus acting as a mediator of insulin resistance and β-cell dysfunction. As a result of the STZ action, β cells undergo the destruction by necrosis.

The pancreas of STZ-induced diabetic rats showed a many pathological changes like a reduction in β-cell number of the islet of Langerhans, beside hemorrhage, arteriosclerosis, and inflammations in both islet and pancreatic acini as well as loss of normal architecture of pancreatic β-cells without any indication of recovery compared to normal tissue. The rats treated with PA flower ethanolic extracts showed significant dose-dependent improvement in cellular architecture as observed by the restoration of the normal cellular population size of islets with hyperplasia, preserved islet cells, with minimal reduction in the size of pancreatic islets; atrophied islets of Langerhans, a slight hemorrhage in the islets of beta cells, a recovery from necrosis in the pancreatic acini, pancreatic morphologies resembling those of normal rats.

This improvement by PA flower ethanolic extracts treatments in the same line with many studies which have indicated that quercetin in PA has the capacity to prevent pancreatic injury, promoting regeneration of the pancreatic islets and increasing its ability to maintain normal blood glucose levels in diabetes-induced rats [57].

### Expected mechanisms for using of PA as complementary and alternative therapy for diabetes mellitus

**Fig. 2:**
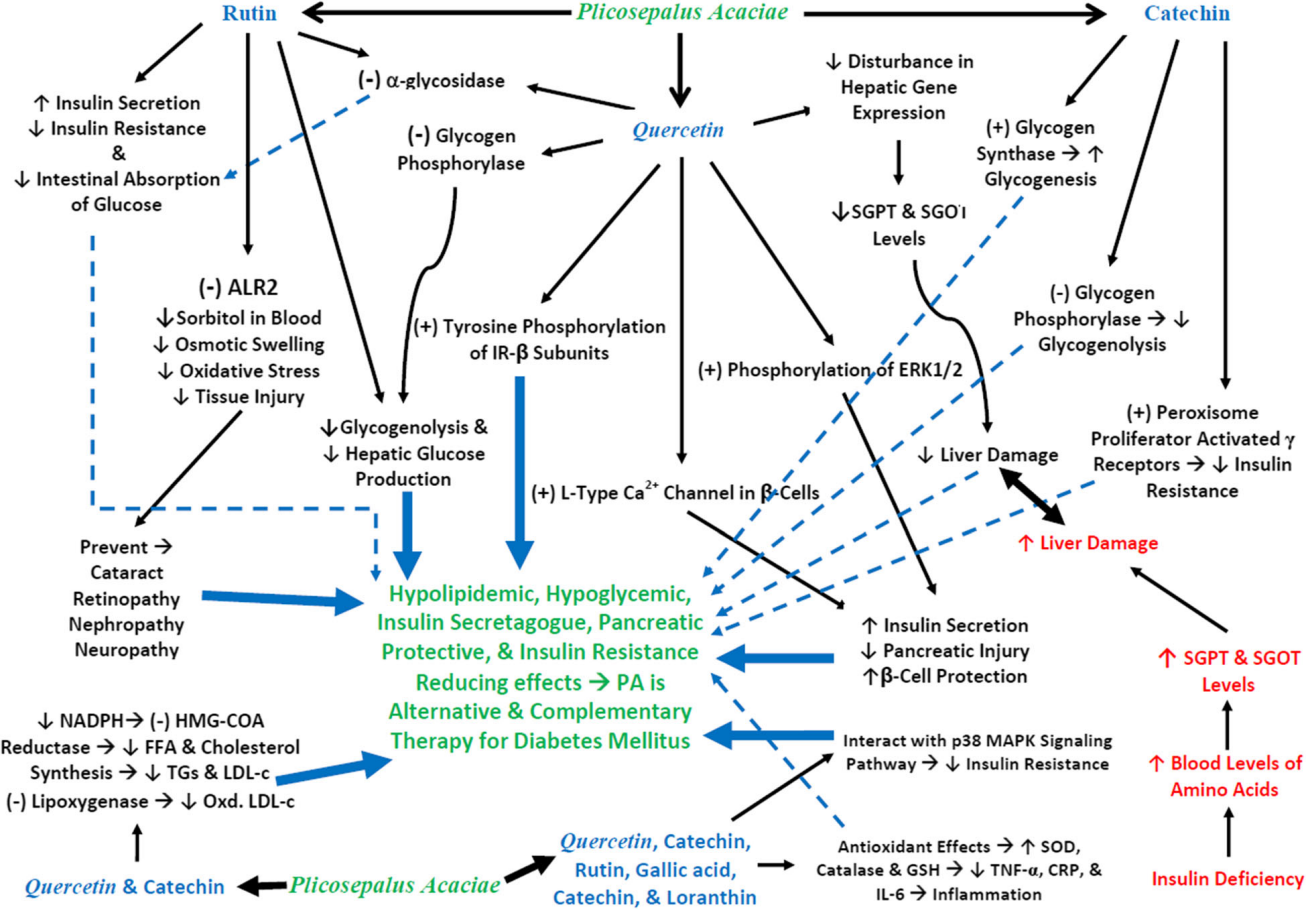
The diagrammatic illustration of PA mechanisms as alternative and complementary therapy for diabetes mellitus. (−) = Inhibition, (+) = Activation, ↑ = Increase, ↓ = Decrease, CRP = C-reactive protein, TNF-α = tumor necrosis factor-alpha, IL-6 = interlukin-6, SGPT = serum glutamate pyruvate transaminase, SGOT = serum glutamate oxaloacetate transaminase, ALR2 = aldose reductase 2, FFA = free fatty acid, TGs = triglycerides, Oxd. LDL-c = oxidized low-density lipoprotein cholesterol, NADPH = reduced nicotinamide adenine dinucleotide phosphate, GSH = reduced glutathione, SOD = superoxide dismutase, MAPK = mitogen activated protein kinase, IR-β = insulin receptor β, ERK1/2 = extracellular signal regulated kinase1/2

Many hypothesis for hypoglycemic mechanisms of PA have been suggested including; 1- potentiating the insulin effect in plasma by increasing either the pancreatic secretion of insulin from the remnant β-cells of the islets of Langerhans, 2- decreasing the hepatic glucose production, inhibiting intestinal glucose absorption or correcting insulin resistance [29], 3- antioxidant activity of its contents of rutin and gallic acid contents [58], and 4- regenerating effect of quercetin (3,5,7,3′,4′-pentahydroxyflavone) on pancreatic islets, preserving the integrity of pancreatic β -cells and normalizing glucose tolerance tests [59]. However, possibilities of other mechanisms to exert the hypoglycemic effect cannot be ruled out (Fig. 2).

#### Effect of PA flower on β-cells, insulin release and insulin resistance

The protection of pancreatic β-cells from inflammation and oxidative stress is essential in preventing or delaying the onset of the disease in at-risk subjects or improvement of diabetic complications [60]. Therefore, the PA flower ethanolic extract acts as antidiabetic by their antioxidant, anti-inflammatory, and repairing effects on β –cells protecting it from further degeneration.

This mechanism confirmed by many studies that stated that quercetin (a flavonoid in the plant) has antioxidant properties, which brings about the regeneration of the pancreatic islets, increases insulin release, and suppresses postprandial hyperglycemia in streptozocin-induced diabetic rats; thus, exerting its beneficial antidiabetic effect [61]. The induction of insulin secretion by quercetin due to its protective effect on β-cell function and antioxidant activity, was confirmed by studies on INS-1 β cell line, where quercetin able to phosphorylate and activates extracellular signal-regulated kinases 1/2 (ERK1/2), which play a major role in the insulin-secreting and β -cell-protecting effects [62]. Additionally, quercetin induces insulin secretion by activation of L-type calcium channels in the pancreatic β-cells [63].

In addition, rutin, another PA constituent, which significantly enhances the insulin release, decreases intestinal glucose absorption, decreases hepatic glucose output, induces the peripheral insulin action and affects mediators of insulin resistance [64]*.*

In general, the biological actions of polyphenols have not been attributed only to their antioxidant properties, but also to their interactions with specific proteins of intracellular signaling cascades, especially the p38 Mitogen-activated protein kinase (MAPK) signaling pathways to decrease insulin resistance [65] (Fig. 2).

#### Effect of the PA flower active constituents on metabolic enzymes

Quercetin inhibits α-glycosidase [61] and activates the tyrosine phosphorylation of insulin receptor-β subunit (IR-β) [66]. According to molecular docking studies, quercetin inhibits glycogen phosphorylase, which leads to the inhibition of glycogenolysis, reduced hepatic glucose production, and lowers blood glucose, thereby exhibiting high potential as a new treatment for type 2 diabetes [67].

Prolonged exposure to chronic hyperglycemia in diabetes can lead to various complications affecting the cardiovascular, renal, neurological, and visual systems [68]. Aldose reductase (ALR2 or AKR1B1; EC: 1.1.1.21) is the first rate-limiting enzyme in the polyol pathway that reduces glucose to sorbitol with NADPH as a cofactor [69]. Accumulation of sorbitol leads to osmotic swelling, changes in membrane permeability, and oxidative stress, which manifest as tissue injury [70]. Rutin is a plant constituent that inhibits the activity of aldose reductase (ALR2), an enzyme normally present in the eye and elsewhere in the body. It is also a competitive inhibitor for α-glycosidase and inhibited glucose absorption [71, 72]. Thus, rutin has a therapeutic value in preventing the onset and/or progression of diabetes and its associated complications, such as cataract, retinopathy, nephropathy, and neuropathy. Meanwhile, catechin is a plant constituent that increases glycogen synthase activity and decreases glycogen phosphorylase activity (i.e., increased glycogenesis and decreased glycogenolysis) [49] (Fig. 2).

## Conclusion

The current study provided a novel strategy and a scientific mechanism of action for the use of PA flower as a prophylactic and an alternative medicine in the treatment of diabetes mellitus due to its antioxidant, anti-inflammatory, hypolipidemic, hypoglycemic and insulin secretagogue effects. Its protective and regenerative effect on pancreatic β cells was attributed to activation of β- cells signaling, which led to improved glucose and lipid metabolism.

## Supplementary information

**Additional file 1 **Figure 1. Histological effects of normal saline [B, C, & D], PA flower ethanolic extract (150 mg/kg) [E, F, & G], PA flower ethanolic extract (300 mg/kg) [H, I, & J] and Metformin (150 mg/kg) [K, L, & M] treatments on pancreatic sections in streptozotocin induced diabetic rats in comparison with non-diabetic healthy pancreatic section [A].

**Additional file 2 **Qualitative phytochemical screening of flower extracts; Qualitative chemical detection of flavonoids, glycosides and simple phenols in all *Plicosepalus acacia* flower extracts.

**Additional file 3.** HPTLC analysis for quantitative separation of rutin, quercetin and gallic acid from the ethanolic, ethylacetate, aqueous, chloroform, and n-hexane extracts of *Plicosepalus acacia* flower.

## Data Availability

All data generated or analyzed during this study are included in this published article.

## Abbreviations

PA: *Plicosepalus Acaciae*
HBA1c%: Glycated hemoglobin percentage
SOD: Superoxide dismutase
GSH: Reduced glutathione
MDA: Malonaldehyde
NO: Nitric oxide
TGs: Triglycerides
Tc: Total cholesterol
LDL-c: Low density lipoprotein cholesterol
VLDL: Very LDL
HDL-c: High DLc
SGPT: Serum glutamic pyruvic transaminase
SGOT: Serum glutamic oxaloacetic transaminase
CRP: C-reactive protein
TNF-α: Tumor necrosis factor-α
IL-6: And interleukin-6
ROS: Reactive oxygen species
STZ: Streptozotocin
GP: Glycogen phosphorylase
HMG-CoA reductase: β-Hydroxy β-methyl glutaryl CoA reductase
ALR2: Aldose reductase 2
ARI: Aldose reductase inhibitor
IR-β subunits: Insulin receptor β subunits
p38 MAPK: p38 mitogen activated protein kinase
ERK1/2: Extracellular signal regulated kinase1/2
