# Supplementary material for: Mechanism of antidiabetic effects of Plicosepalus Acaciae flower in streptozotocin-induced type 2 diabetic rats, as complementary and alternative therapy
Source: BMC Complement Med Ther. 2020 Sep 23;20:290. doi: 10.1186/s12906-020-03087-z (PMC7509926; doi:10.1186/s12906-020-03087-z)
Supplement: Supplementary file 1 — Additional file 1 Figure 1. Histological effects of normal saline [B, C, & D], PA flower ethanolic extract (150 mg/kg) [E, F, & G], PA flower ethanolic extract (300 mg/kg) [H, I, & J] and Metformin (150 mg/kg) [K, L, & M] treatments on pancreatic sections in streptozotocin induced diabetic rats in comparison with non-diabetic healthy pancreatic section [A]. [file 12906_2020_3087_MOESM1_ESM.doc]

**Fig. 1 Histological effects of normal saline [B, C, & D], PA flower ethanolic extract (150 mg/kg) [E, F, & G], PA flower ethanolic extract (300 mg/kg) [H, I, & J] and Metformin (150 mg/kg) [K, L, & M] treatments on pancreatic sections in streptozotocin induced diabetic rats in comparison with non-diabetic healthy pancreatic section [A].** These figures were showed a normal architecture of pancreatic acini & normal islet of Langerhans in non-diabetic control [**A**], while a reduction in β-cell number i.e., destruction of the islet of Langerhans (*****) [**B**] with a necrosis in a pancreatic acini (arrows) [**C**], fatty changes in pancreatic acini (interstitial vaculation 🡪 arrow) **[D**] in pancreas of normal saline treated diabetic rats. Moreover, there was a reduction in the size of pancreatic islets; atrophied islets of Langerhans (arrows) [**E**], with a slight hemorrhage in the islets of beta cells (red color) [**F& G**] in pancreas of PA (150 mg/kg) treated diabetic rats. A reduction in the size of islets; atrophied islet of Langerhans (arrow) [**H**], with a slight hemorrhage in the pancreatic acini (arrow) [**I**], and a recovering from necrosis in the pancreatic acini [**J**] of PA (300 mg/kg) treated diabetic rats, while a hemorrhage in a pancreatic acini congestion (red color) [**K**], with a reduction in number of beta cells, hemorrhage [**L**], and slight minimal necrotic cells (arrows) [**M**] in the pancreatic acini of Metformin (150 mg/kg) treated diabetic rats (H & E 400X; Scale bar = 50 µm).
