# Supplementary material for: Mechanism of antidiabetic effects of Plicosepalus Acaciae flower in streptozotocin-induced type 2 diabetic rats, as complementary and alternative therapy
Source: BMC Complement Med Ther. 2020 Sep 23;20:290. doi: 10.1186/s12906-020-03087-z (PMC7509926; doi:10.1186/s12906-020-03087-z)
Supplement: Supplementary file 2 — Additional file 2 Qualitative phytochemical screening of flower extracts; Qualitative chemical detection of flavonoids, glycosides and simple phenols in all Plicosepalus acacia flower extracts. [file 12906_2020_3087_MOESM2_ESM.doc]

**Qualitative chemical detection of flavonoids, glycosides and simple phenols in *Plicosepalus acacia* flower extracts.**

| **Extraction method** | **Simple phenol tests** | **Glycosides test** | **Flavonoids test** |
| --- | --- | --- | --- |
| **Ethanol** | FeCl_3_ (+++)  Lead acetate (++)  Dil.HNO_3_ (+) | Borntrager (-)  Legal (++)  Keller-Killiani (+++) | FeCl_3_ (+++)  Lead acetate (+++)  Shinoda (+++)  Sodium hydroxide (+++) |
| **Ethyl Acetate** | FeCl_3_ (-)  Lead acetate (-)  Dil.HNO_3_ (-) | Borntrager (-)  Legal (+)  Keller-Killiani (++) | FeCl_3_ (-)  Lead acetate (-)  Shinoda (+)  Sodium Hydroxide (++) |
| **Aqueous** | FeCl_3_ (-)  Lead acetate (-)  Dil.HNO_3_ (-) | Borntrager (-)  Legal (-)  Keller-Killiani (+) | FeCl_3_ (-)  Lead acetate (-)  Shinoda (-)  Sodium Hydroxide (+) |
| **Chloroform** | FeCl_3_ (-)  Lead acetate (-)  Dil.HNO_3_ (-) | Borntrager (-)  Legal (-)  Keller-Killiani (-) | FeCl_3_ (-)  Lead acetate (-)  Shinoda (-)  Sodium Hydroxide (-) |
| **N-Hexane** | FeCl_3_ (-)  Lead acetate (-)  Dil.HNO_3_ (-) | Borntrager (-)  Legal (-)  Keller-Killiani (-) | FeCl_3_ (-)  Lead acetate (-)  Shinoda (+)  Sodium Hydroxide (-) |

Sign (+) indicates present and sign (−) indicates absent.
