# Supplementary material for: Mechanism of antidiabetic effects of Plicosepalus Acaciae flower in streptozotocin-induced type 2 diabetic rats, as complementary and alternative therapy
Source: BMC Complement Med Ther. 2020 Sep 23;20:290. doi: 10.1186/s12906-020-03087-z (PMC7509926; doi:10.1186/s12906-020-03087-z)
Supplement: Supplementary file 3 — Additional file 3. HPTLC analysis for quantitative separation of rutin, quercetin and gallic acid from the ethanolic, ethylacetate, aqueous, chloroform, and n-hexane extracts of Plicosepalus acacia flower. [file 12906_2020_3087_MOESM3_ESM.doc]

**Quantitative separation by HPTLC of rutin, quercetin and gallic acid from the ethanolic, ethylacetate, aqueous, chloroform, and n-hexane extracts of *Plicosepalus acacia* flower.**

| **Type of the sample** | **Concentration of rutin (366 nm)** | | | **Concentration of quercetin (280 nm)** | | | **Concentration of gallic acid (254 nm)** | | |
| --- | --- | --- | --- | --- | --- | --- | --- | --- | --- |
|  | **R_f_** | **Area** | **g%** | **R_f_** | **Area** | **g%** | **R_f_** | **Area** | **g%** |
| **PA Ethanolic Extract** | 0.39 | 14600.4 | 3.147 | 0.79 | 1750.6 | 6.515 | 0.82 | 927.5 | 0.701 |
| **PA Ethyl Acetate Extract** | 0.42 | 4860.6 | 1.047 | 0.82 | 299.1 | 1.113 | --- | --- | --- |
| **PA Aqueous Extract** | 0.43 | 2804.4 | 0.604 | 0.83 | 140.6 | 0.560 | --- | --- | --- |
| **PA Chloroform Extract** | --- | --- | --- | --- | --- | --- | --- | --- | --- |
| **PA -Hexane Extract** | --- | --- | --- | 0.84 | 100.9 | 0412 | --- | --- | --- |
